# Association of Polygenic Variants Involved in Immunity and Inflammation with Duodenal Ulcer Risk and Their Interaction with Irregular Eating Habits

**DOI:** 10.3390/nu15020296

**Published:** 2023-01-06

**Authors:** Sunmin Park, Meiling Liu, Shaokai Huang

**Affiliations:** 1 Department of Food and Nutrition, Obesity/Diabetes Research Center, Hoseo University, Asan 31499, Republic of Korea; 2 Department of Bioconvergence, Hoseo University, Asan 31499, Republic of Korea

**Keywords:** duodenal ulcer, polygenic risk score, rare meat eating, smoking, irregular eating

## Abstract

Genetic and environmental factors are associated with developing and progressing duodenal ulcer (DU) risk. However, the exact nature of the disease pathophysiology and the single nucleotide polymorphism (SNP)—lifestyle interaction has yet to be determined. The purpose of the present study was to examine the SNPs linked to DU risk and their interaction with lifestyles and diets in a large hospital-based cohort of Asians. Based on an earlier diagnosis, the participants were divided into the DU (case; n = 1088) and non-DU (control, n = 56,713) groups. The SNP associated with DU risk were obtained from a genome-wide association study (GWAS), and those promoted genetic impact with SNP–SNP interactions were identified with generalized multifactor dimensionality reduction analysis. The interaction between polygenic risk score (PRS) calculated from the selected genetic variants and nutrient were examined. They were related to actin modification, immune response, and cell migration by modulating leucine-rich repeats (LRR) domain binding, Shaffer interferon regulatory factor 4 (IRF4) targets in myeloma vs. mature B lymphocyte, and Reactome runt-related transcription factor 3 (RUNX3). Among the selected SNPs, rs11230563 (R225W) showed missense mutation and low binding affinity with different food components in the autodock analysis. Glycyrrhizin, physalin B, janthitrem F, and casuarinin lowered it in only wild CD6 protein but not in mutated CD6. Plastoquinone 8, solamargine, saponin D, and matesaponin 2 decreased energy binding affinity in mutated CD6 proteins. The PRS of the 5-SNP and 6-SNP models exhibited a positive association with DU risk (OR = 3.14). The PRS of the 5-SNP PRS model interacted with irregular eating habits and smoking status. In participants with irregular eating habits or smokers, DU incidence was much higher in the participants with high PRS than in those with low PRS. In conclusion, the genetic impact of DU risk was mainly in regulating immunity, inflammation, and actin modification. Adults who are genetically susceptible to DU need to eat regularly and to be non-smokers. The results could be applied to personalize nutrition.

## 1. Introduction

A peptic ulcer is a lesion found in the stomach and duodenum. It shows a denuded mucosa that extends through the muscularis mucosa into the deeper layers of the wall, such as the submucosa or muscularis propria. According to the anatomical location, they may be termed gastric (GU) or duodenal ulcers (DU) [1]. The most common type of peptic ulcer is DU. Their prevalence is reported to be 0.1–0.19% worldwide and is higher among the Asian population [1]. Although the figures vary in different studies, the prevalence of GU and DU is 3.3% and 2.1%, respectively, in Korea [1]. A peptic ulcer can occur at all ages, but it is more common in middle-aged adults [1]. The symptoms of peptic ulcers are varied, with mild to severe epigastric pain usually felt in the upper abdomen just below the breastbone accompanied by aches, burning, or soreness. Taking an antacid to reduce gastric acid usually relieves the pain, but it commonly recurs after several weeks.

Peptic ulcers occur when the healthy defense and mucosal repair mechanisms of the gastric or duodenal walls are weakened. In the past, peptic ulcers were believed to occur due to the excessive secretion of gastric acid and digestive enzymes that damaged the mucosal lining [2]. However, recently, *Helicobacter pylori* (*H. pylori*) infection and taking non-steroidal anti-inflammatory drugs (NSAIDs), including pyrine drugs, in addition to smoking and alcohol intake, are recognized as the primary risk factors for the occurrence of peptic ulcers [3]. The risk factors are also involved in increased reactive oxygen species (ROS) production in the stomach and intestines to exacerbate ulceration by altering muscular alteration [4]. The increased ROS contributes to the progress of PU into gastric carcinogenesis by impairing the immune system and elevating inflammation [5]. Approximately 50–70% of patients with DU and 30%–50% of patients with GU have an *H. pylori* infection [1]. Moreover, the recurrence rate of peptic ulcers decreases with anti-*H. pylori* treatment. In addition, gastritis and duodenitis, often caused by *H. pylori*, can progress into a peptic ulcer. Pain relievers such as antipyretics, analgesics, and anti-inflammatory drugs cause DU by blocking the production of prostaglandins that regulate the regeneration and function of the duodenal mucosal cell layer [6]. The relationship between food and nutrient intake and peptic ulcers has been studied, but it is still controversial. Despite being clubbed together due to similar symptomatology, GU and DU differ in their pathophysiology. In GU, morphological changes occur in the gastric mucosa, similar to the changes in gastric cancer, accompanied by atrophic gastritis and intestinal metaplasia. Peptic ulcer, including DU, is generally characterized by low gastric acid secretion. However, DU is often associated with high secretion of gastric acid, and patients with DU are only half as likely to develop stomach cancer compared to those without DU.

DU is linked to genetic and environmental factors. About 60% of children with DU have a family history of peptic ulcers, indicating a high genetic impact. Most studies investigating their genetic effect have determined the relationship of the host genes with *H. pylori* [7,8]. The host’s genetic vulnerability to *H. pylori* infection is linked to the immunity and inflammation-related genes such as tumor necrosis factor (*TNF*)-α, Toll-like receptor-4 (*TLR-4*), interleukin (*IL*)-6, angiotensin-converting enzyme (*ACE*)1/D [7,8] and oxidative stress-related genes such as catalase, Mn-superoxide dismutase (*SOD*), and glutathione reductase-1 (*GPX1*) [9]. Using the UK biobank data, eight independent loci for peptic ulcer are revealed to be located at or near ABO, mucin (*MUC*)*1*, *MUC6*, fucosyltransferase 2 (*FUT2*), prostate stem cell antigen (*PSCA*), caudal type homeobox 2 (*CDX2*), gastrin (*GAST*), and cholecystokinin B receptor (*CCKBR*) genes. These genes are related to susceptibility to *H. pylori* infection, mucosal damage, gastric acid secretion, and gastrointestinal mobility [10]. Additionally, genetic variants of glutathione S-transferase theta 1 (*GSTT1*) and prostate stem cell antigen (*PSCS*) related to DU were inversely associated with gastric cancer risk in Japanese [11] and Indians [12], respectively. Therefore, there could be a genetic association between DU, immunity, and gastric cancer.

However, studies have yet to be conducted to understand the interaction between genetic differences and lifestyles for DU risk. In the present study, we focused on examining the genetic variants associated with DU and their interaction with environmental factors influencing DU risk. This study was performed on a large hospital-based cohort of Asian subjects.

## 2. Materials and Methods

### 2.1. Participants

Volunteers aged over 40 who visited the metropolitan hospitals participated in a hospital-based cohort as part of the Korean Genome and Epidemiology Study (KoGES) during 2010–2014 [13]. The institutional review board (IRB) of the Korean National Institute of Health and Hoseo University approved the KoGES (KBP-2015-055) and the current study (HR-034-01), respectively. All participants signed a written informed consent form.

### 2.2. DU Diagnosis

Participants previously diagnosed with DU by a doctor were included in the DU group in the study. Those undiagnosed with DU were considered as the non-DU group (control). Their current treatments and remission status were checked, and the diagnosis was verified through a survey.

### 2.3. Characteristics of the Participants by Interview and Biochemical Assays

Participant’s age, gender, income, education, and area of residence, were collected using the survey questionnaires. Their height and weight were measured wearing light clothes and bare feet, as described previously [14,15]. The appendicular skeletal muscle mass and fat mass were estimated indirectly using the prediction model generated by the XGBoost algorithm from the Ansan/Ansung cohort, where the mass was measured by the Inbody^®^ body composition analyzer (Cheonan, Korea) [16]. A doctor measured the participant’s blood pressure in the sitting position thrice using a blood pressure monitor, and the average diastolic blood pressure (DBP) and systolic blood pressure (SBP) were calculated for the study. The participants were queried about any previous diagnosis of inflammatory diseases, including bronchitis, asthma, arthritis, osteoporosis, allergy, periodontitis, and gastritis.

After fasting for more than 12 h, serum was separated from blood collected from the vacutainer without blood coagulant and plasma from the vacutainers containing ethylenediaminetetraacetic acid (EDTA) and heparin. Fasting plasma glucose and glycosylated hemoglobin (HbA1c) concentrations were measured using a Hitachi 7600 Automatic Analyzer (Hitachi, Tokyo, Japan) and an automatic analyzer (ZEUS 9.9; Takeda, Tokyo, Japan), respectively. Biochemical parameters in the serum were determined using a Hitachi 7600 Automatic Analyzer. The parameters were as listed as follows: total cholesterol, high-density lipoprotein cholesterol (HDL-C), triglycerides (TG), total bilirubin, and creatinine concentrations, and alanine aminotransferase (ALT) and aspartate aminotransferase (AST) activities. Serum high-sensitive C-reactive protein (hs-CRP) concentrations were measured using a high-sensitive enzyme-linked immunosorbent assay (ELISA) kit (Thermofisher, Waltham, MA, USA). White blood cells (WBC) were counted with an automated hematology analyzer (Beckman Coulter, Brea, CA, USA) using the EDTA-treated blood. Insulin resistance was predicted using a machine learning prediction model made with the homeostatic model assessment of insulin resistance (HOMA-IR) in the An-san/Ansung cohort from the Park et al. study [17].

From the health survey, the daily alcohol intake (g/day) was calculated by multiplying its consumption frequency by the amounts consumed [18]. Participants who had smoked at least 20 cigarettes in the past six months were considered current smokers, and those who smoked more than 20 cigarettes but had not smoked for at least six months were former smokers [18]. Coffee intake was estimated with a similar method of alcohol intake. Regular exercise was defined as more than 150 min of moderate physical activity per week.

### 2.4. Usual Food Intake and Dietary Patterns Using and Dietary Patterns by Principal Component Analysis (PCA)

Participants were asked about their dietary habits in the questionnaires. Their usual food intake during the last 12 months was determined with a semi-quantitative food frequency questionnaire (SQFFQ) containing 106 food items Koreans commonly consume. The reproducibility and accuracy of the SQFFQ were validated using three-day food records during the four seasons [19]. The SQFFQ consisted of food frequencies such as never or seldom, once per month, two to three times per month, once or twice weekly, three or four times weekly, five or six times weekly, daily, twice daily, and ≥3 times daily. The food amount was classified as less than, equal to, or more than portion size, and the designated portion size of each food was visualized with a photograph. The daily intake of 106 food items (g/day) was calculated by multiplying the portion sizes by the median of the daily-consumed frequencies. The nutrient intake was estimated from the daily food intake using the Can-Pro 2.0 nutrient assessment program designed by the Korean Nutrition Society (KNS).

The dietary patterns were stratified with the PCA of the 30 predefined groups based on eigenvalues > 1.5 [20]. The dietary patterns were classified using the orthogonal rotation procedure (varimax), and food groups with ≥0.40 factor-loading values were selected [21]. The dietary patterns were divided into four types named as the Korean balanced diet (KBD), plant-based diet (PBD), Western-style diet (WSD), or rice-based diet (RBD).

The dietary inflammatory index (DII) was assessed using the prediction equation by multiplying food and nutrient intakes by their dietary inflammatory weights, as reported previously [22]. DII was calculated as the sum of the individual scores divided by 100.

### 2.5. Genotyping Using a Korean Chip

Genotyping was conducted with the genomic DNA separated from whole blood using a Korean Chip (Affymetrix, Santa Clara, CA, USA), including SNPs involved in the disease-related single nucleotide polymorphisms (SNPs) in Koreans [23]. The genotypes were imputed with the Korean HapMap data or 1000-genome sequence [24], and their accuracy was determined using a Bayesian learning algorithm for Robust General Linear Models (RGLMs) [25]. Their exclusion criteria were <98% genotyping accuracy, ≥30% heterozygosity, ≥4% missing genotype call rate, and no gender bias. After the genome-wide association study (GWAS) for DU risk, the genetic variants with *p* < 0.05 of the Hardy–Weinberg Equilibrium (HWE) and <1% minor allele frequency (MAF) were excluded [25]. A Manhattan plot was displayed with the negative logarithms of the *p* value of the genetic variants from the GWAS for DU risk. A quantile-quantile (Q-Q) plot represents a probability plot for the actual data distribution vs. the theoretical data distribution of the *p*-values of the genetic variants. The Q-Q plot displayed the quantile distribution of observed and expected *p*-values as the *y*-axis and the *x*-axis, respectively. The lambda value of the Q-Q plot indicated the goodness of fit of the actual *p* values to the theoretical ones. When the lambda value of the Q-Q plot was close to one, the genotypes were correctly conducted. The plots were generated from the Snp2gene of the functional mapping and annotation of genetic associations (FUMA) website. The pathways involved in the genetic variants for DU risk were selected with *p* value with Bonferroni correction <0.05 using the MAGMA gene-set analysis in the Snp2gene of the FUMA web application (https://github.com/Kyoko-wtnb/FUMA-webapp/ accessed on 10 December 2022).

### 2.6. Selection of the Genetic Variants for DU Risk and SNP-SNP Interaction Model

**Figure 1:**
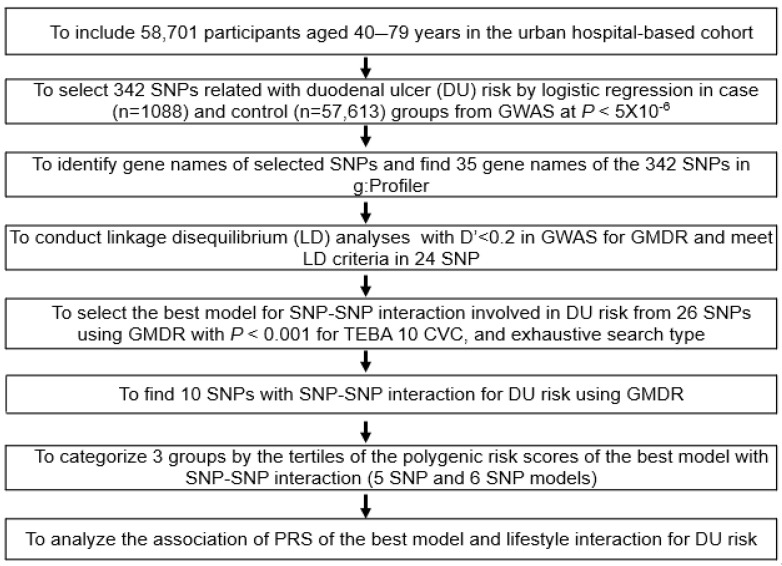
Flow chart to generate polygenic risk score (PRS) linked to duodenal ulcers by SNP-SNP interaction and its interaction with lifestyle factors. SNP: Single nucleotide polymorphisms.

Figure 1 presents the best genetic model selection procedure with genetic variant-genetic variant interactions. In the urban hospital-based cohort, genetic variants for DU risk were chosen from GWAS of the DU (n = 1088) and non-DU groups (n = 57,613). The 342 genetic variants were selected to meet the criteria of *p* < 5 × 10^−6^, and the matched gene names of the genetic variants were found on the g:Profiler website (https://biit.cs.ut.ee/gprofiler/snpense accessed on 10 December 2022). The genetic variants with linkage disequilibrium (LD) relations (D’ ≥ 0.2) were found using Haploview 4.2 in PLINK, and they were excluded due to providing identical genetic information for DU risk. From the 252 genetic variants matched with gene names, 24 genetic variants with identified gene names remained after excluding the genetic variants with D’ ≥ 0.2.

Genetic variants interacting with each other to promote DU risk were selected from 24 SNPs involved in DU risk using generalized multifactor dimensionality reduction (GMDR). The best genetic variant model was assigned to satisfy *p* < 0.001 in the sign test of testing balanced accuracy (TEBA) and ten-fold cross-validation consistency (CVC) in the exhaustive search type [26]. Two different sets of covariates were used for the analysis: Covariates of set 1 included age, gender, residence area, survey year, body mass index (BMI), education, and income. The covariates of set 2 contained set 1 plus energy intake, alcohol, and coffee consumption, physical activity, and smoking status.

The polygenic risk score (PRS) for the optimal genetic model was generated by adding the number of the risk alleles in each genetic variant with the selected genetic variant-genetic variant interaction model. If the risk allele of a genetic variant was G, the genetic scores of “GG”, “AG”, and “AA” were 2, 1, and 0, respectively. Among the models to meet the sign test of TEBA and CVC, the optimal model was selected by comparing the adjusted odds ratio (OR). The PRS of the optimal model was stratified into three categories viz. Low-PRS (PRS < 4), Middle-PRS (PRS = 4 and 5), and High-PRS (PRS > 5). Adjusted OR and 95% CI of DU risk were calculated with the PRS groups, and the best model was selected, which had a smaller number of SNPs and a bigger adjusted OR.

### 2.7. Molecular Docking of Food Compounds and Targets of Genes Related to DU

Among the corresponding genes of the selected 10 genetic variants, genes with missense genetic variants were used for changing binding affinity with food components. The changing binding affinity indicated that the catalytic activity of the genes would be changed. The wild-type protein structures of the genes with missense genetic variants were generated in the *Protein Data Bank* (PDB) format from the Iterative Threading Assembly Refinement (I-TASSER) website. The mutated gene was made by changing the amino acid corresponding to the genetic variants in the Swiss-PBD Viewer (SPDBV) program. Primary ligands were isolated from water molecules and hetero-macromolecules, such as whole protein, using the pleomorphic analysis methodology (PyMOL) software (DeLano Scientific LLC, USA) [27]. The PDB structure was converted into a PBD, partial charge (Q), and atom type (T; PDBQT; AutoDock format) lattice format using AutoDock Tools 1.5.6 (Molecular Graphics Laboratory, The Scripps Research Institute, Jupiter, FL, USA) [27]. The active sites in the protein were searched using the proteins plus website (https://proteins.plus/, accessed on 3 March 2022), and the active functional pockets with mutated sites were selected for molecular docking.

Food compounds (n = 20,000) were downloaded from the fooDB website (https://foodb.ca/, accessed on 4 January 2022) and made into PDBQT files using AutoDock Tools 1.5.6. The active pockets, including the mutated site, acted as receptors for molecular docking with the food components. After each compound was docked, the compounds with an energy binding affinity of less than −10 were chosen [28]. The lower binding affinity indicated better binding affinity with the active site.

### 2.8. Molecular Dynamics Simulation (MDS)

MDS was used to examine the conformational changes of the protein structure with a variant relative to the native conformation. It also detected changes in protein phenotypes to confirm the devastating consequences of computationally predicted disease-associated mutations. After the top docking poses of all investigated compounds were added, the simulations were performed on pure protein receptors and docked complexes separately by the DS software. The Chemistry at Harvard Macromolecular Mechanics (CHARMM) force field was added to each molecular structure prepared by “Simulation”, and the protein was solvated by “Solvation”.

The “Standard Dynamics Cascade” set the molecular dynamics simulation parameters for the protein added to the solvent system. The parameters were set to default values, except that the ramp-up time, equilibration time, simulation sampling time, and simulation step size were set to 40 ps, 400 ps, 10,000 ps, and 2 fs, respectively. The root mean square deviation (RMSD), root mean square fluctuations (RMSF), and hydrogen bond values were analyzed after the 10 ns simulation.

### 2.9. Statistical Analysis

The statistical analysis was conducted using SAS (version 9.3; SAS Institute, Cary, NC, USA) [29]. The 58,701 participants were the appropriate sample size to examine the significant differences of the genetic variants for the disease with about 2% prevalence at a statistical significance at α = 0.05, β = 0.99, and an odds ratio of 1.2 in the logistic regression analysis using a G-power calculator. The results of categorical variables were analyzed to show the frequency distributions, and their statistical differences between the DU and the non-DU groups were assessed using the chi-square test. The adjusted means with standard deviations of the continuous variables were calculated with the adjustment of covariates. The statistical differences between the genders and the DU/non-DU groups were determined using a two-way analysis of covariance (ANCOVA) [29]. If ANCOVA was significant at *p* < 0.05, multiple comparisons were conducted using Tukey’s test.

The adjusted odds ratios (ORs) and 95% confidence intervals (CI) of DU were calculated in each metabolic parameter using logistic regression analysis after adjustment for covariates. Two different models with different covariates were applied to calculate adjusted ORs: covariates of model 1 included age, gender, survey year, residence area, body mass index (BMI), income, and education, and those of model 2 were the covariates of model 1 plus energy intake, alcohol and coffee consumption, physical activity, and smoking status.

Two-way ANCOVA with main effects and interaction terms was applied to analyze the interactions between DU and lifestyles after each lifestyle was categorized into the low or high groups according to the 30th percentiles of each variable or the dietary reference intake [30]. Adjusted ORs and 95% CI of DU with PRS were also determined using adjusted logistic regression analysis with covariates in the low and high groups of each lifestyle-related parameter. The proportion of DU patients was calculated according to the PRS groups using the χ^2^ test in the low and high groups of each lifestyle-related parameter.

## 3. Results

### 3.1. General Characteristics According to Their Gender and DU

The participants with DU were older than those without DU, and men had more DU than women (*p* < 0.0001; Table 1). The BMI and waist circumferences were lower in the DU group than in the non-ulcer group in both genders and were inversely associated with DU risk (*p* < 0.001). Serum glucose concentrations were lower in the DU than in the non-ulcer groups in both genders but not the HbA1c levels (Table 1). The WBC count was higher in men and the DU group and was inversely associated with DU (Table 1). However, there was no significant difference in serum hs-CRP concentrations between the non-DU and DU groups. The incidence of metabolic syndrome did not differ between the groups. The incidence of immune-related diseases such as bronchitis, asthma, arthritis, allergy, gastritis, and periodontitis was higher in the DU group than in the non-ulcer group, regardless of gender. The risk of DU was 2.06–3.34 times higher in patients with these immune-related diseases. Interestingly, the risk of osteoporosis is 1.93 times higher in those with DU.

### 3.2. Dietary Intake and Lifestyles According to Gender and DU

Energy intake was not significantly different between the non-DU and DU groups (Table 2). Among the four dietary patterns, the participants with DU had a lower proportion of WSD intake than those without ulcers, and the WSD was inversely associated with DU. The participants consuming meat cooked less showed a higher incidence of DU than those who did not. Those consuming lower amounts of cooked meats were at a 1.24 times higher risk of DU (Table 2). However, the intake of burnt meats and fried food was not associated with DU risk. The participants with DU had a lower coffee intake than those without DU, and coffee intake was inversely related to DU risk. However, there was no difference in the tea and alcohol intakes between the DU and non-DU groups (Table 2). Interestingly, the number of participants taking multivitamins was lower in the DU group than in the non-DU group, and taking multivitamin showed an inverse association with DU risk (Table 2). Physical activity was not associated with DU risk. As expected, among men, non-smokers had a lower incidence of DU than former and current smokers, and smoking status exhibited a positive association with DU risk (Table 2).

### 3.3. Characteristics of Polygenic Variants Involved in DU Risk

**Figure 2:**
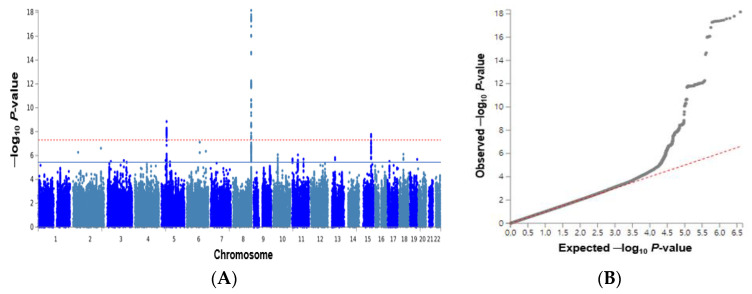
Distribution of genetic variants associated with duodenal ulcers based on the genome-wide association study. (**A**) Manhattan plot of the *p*-value of genetic variants for duodenal ulcer risk. (**B**) Quantile–quantile (Q–Q) plot of observed and expected *p*-values for duodenal ulcer risk.

The Manhattan plot showed the statistical significance of each polymorphism with DU risk in the GWAS. The red lines indicated *p* < 5 × 10^−8^, which was applied with the Bonferroni correction. However, we used a statistical significance of *p* < 5 × 10^−6^ since *p* < 5 × 10^−8^ was conservative, and many genetic variants showed LD relations. Significant genetic variants existed in chromosomes 2, 5, 6, 8, 11, 13, and 15 (Figure 2A). The Q-Q plot showed the deviation of the observed *p* values for genetic variants from their expected *p* values for DU risk. The lambda value representing the genome inflation factor was 1.034, indicating no inflation of the genetic variants associated with DU risk (Figure 2B).

The genetic characteristics of DU-related genetic variants with SNP-SNP interaction are presented in Table 3. They were satisfied with *p* < 5 × 10^−6^ for GWAS with DU, ≥0.01 in MAF, D’ < 0.2 in LD, and *p* ≥ 0.05 in HWE. Ten genetic variants were listed in Table 3 as follows: rs576376935_C-X-C Motif Chemokine Receptor 2 (*CXCR2*), rs77063016_fragile histidine triad diadenosine triphosphatase (*FHIT*), rs10055925_tetratricopeptide repeat domain 33 (*TTC33*), rs2978977_*PSCA*, rs6584283_*LINC01475*, rs11230563_cluster of differentiation 6 (*CD6*) (R225W), rs7309887_inositol 1,4,5-trisphosphate receptor type 2 (*ITPR2*), rs78141015_ DNAJ heat shock protein family (*DNAJC15*), rs111690253_lipopolysaccharide-induced TNF factor (*LITAF*), and rs796980537_fucosyltransferase 2 (*FUT2*). The rs10055925_*TTC33*, rs2978977_*PSCA*, rs11230563_*CD6*(R225W), and rs7309887_*ITPR2* (OR = 0.637–0.813) variants were inversely associated with DU risk, while the other six genetic variants (OR = 1.17–1.95) were positively associated with DU risk. The rs11230563_*CD6* (R225W) variant was located in the gene transcript, while others were in the intron.

### 3.4. Pathways of DU Risk-Related Genetic Variants

The gene ontology (GO) terms and curated gene sets related to the genetic variants associated with DU risk were searched with a MAGMA gene-set analysis. Table 4 displays the pathways involved in the genetic variants associated with DU risk. The biological process in the GO, the actin modification pathway, was associated with the genetic variants associated with DU risk. The potential genes related to actin modification were *CXCR2*, *FHIT*, *CD6*, *ITPR2*, and *DNAJC15*. In the GO of molecular function, the leucine-rich repeats (LRR) domain binding pathway exhibited an association with the genetic variants linked to DU risk. Its related potential genes were *CD6*, *FUT2*, *LITAF*, and *FHIT*. The pathway of Shaffer interferon regulatory factor 4 (*IRF4*) targets in myeloma vs. mature B lymphocytes was associated with DU risk, potentially with the genetic variants of *CXCR2*, *CD6*, *ITPR2*, and *FUT2*. Furthermore, the pathway of the Reactome runt-related transcription factor 3 (runx3) regulates immune response and cell migration, which was linked to DU risk, possibly with the genetic variants of *CXCR2*, *FHIT*, *CD6*, *ITPR2*, and *DNAJC15*.

### 3.5. PRS of Genetic Variants Associated with DU Risk

In the GMDR analysis, after adjusting with covariates, models 2, 5, 6, 7, 8, 9, and 10, as described below, satisfied the criteria of the sign test (*p* < 0.05) for TEBA and CVC (10/10). It indicated that the selected models were the candidates for the best models (Supplementary Table S1). Model 2 included rs2978977_*PSCA* and rs796980537_*FUT2* and model 5 contained *TTC33*_rs10055925, *FUT2*_rs796980537, *LINC01475*_rs6584283, *ITPR2*_rs7309887, and *PSCA*_rs2978977. *CD6*_rs11230563, *FHIT*_rs77063016, *DNAJC15*_rs78141015, *LITAF*_ rs111690253, and *CXCR2*_rs576376935 were added into genetic variants in model 5 to generate models 6, 7, 8, 9 and 10 (Supplementary Table S1).

**Figure 3:**
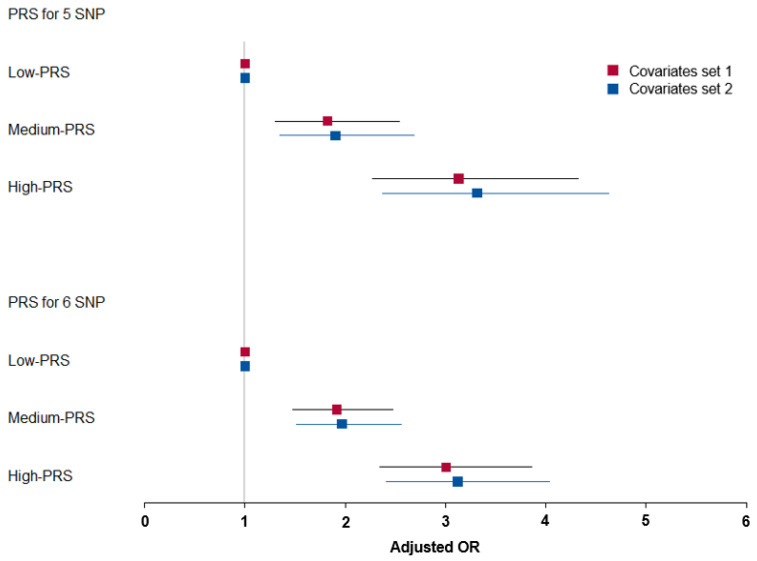
Adjusted odds ratio (ORs) and 95% confidence intervals (CI) of 3-SNP PRS and 6-SNP PRS for duodenal ulcer risk. PRS was generated with the sum of the number of risk alleles in each genetic variant and classified into Low-PRS (0–3), Middle-PRS (4–5), and High-PRS (≥6) in the five-SNP model, respectively. SNP: Single nucleotide polymorphism; PRS: Polygenic risk score.

The PRS of models 5 and 6 exhibited a positive association with DU risk by about three times after adjusting with two sets of covariates (Figure 3). The covariates of set 1 included age, gender, BMI, residence area, education, and income. Those of set 2 contained energy intake, alcohol intake, smoking status, and physical exercise plus covariate set 1. These results suggested that the five-SNP model accounted for DU risk in both covariate sets. However, PRS was not associated with other immune-related and metabolic parameters such as WBC, hs-CRP, BMI, and waist circumference and disease conditions such as metabolic syndrome, bronchitis, asthma, arthritis, allergy, osteoporosis, gastritis, and periodontitis (Supplementary Table S2). 

### 3.6. Energy Binding Affinity with Food Components and the Foods Containing the Food Components

**Figure 4:**
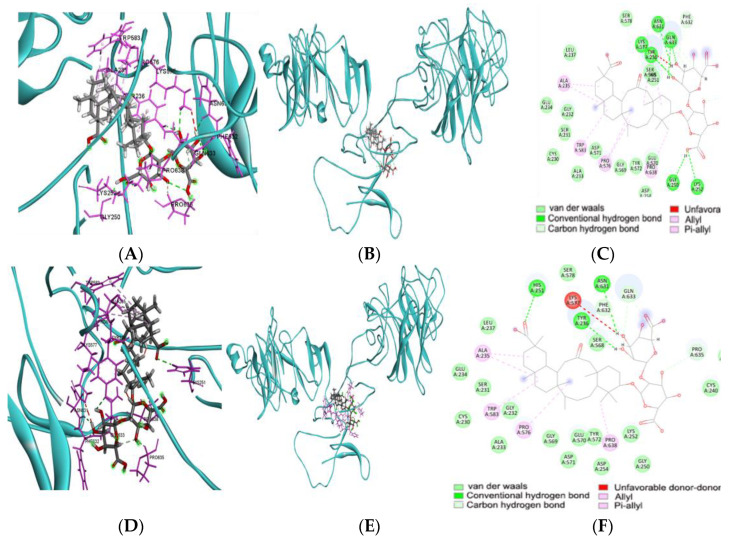
Molecular docking of glycyrrhizin with rs11230563 *CD6* active site (**A**) Active site of the wild type of CD6 protein binding to glycyrrhizin (cartoon model). (**B**) Overall binding glycyrrhizin with the wild type of CD6 protein. (**C**) The wild type of CD6 protein’s active site binds glycyrrhizin with an intermolecular binding force. (**D**) The active site of the mutated type of CD6 protein binding to glycyrrhizin (cartoon model). (**E**) Overall binding glycyrrhizin with the mutated type of CD6 protein. (**F**) The active site of the mutated type of CD6 protein binding glycyrrhizin with intermolecular binding force.

Food components with lower binding affinity improved the wild and mutated CD6 protein activity. Foods containing high levels of these food components are presented in Table 5. Azaspiracid 2 showed low binding affinity (−13.2 kcal/mol) in both wild and mutated types of CD6 protein (Table 5). However, glycyrrhizin, physalin B, janthitrem F, and casuarinin lowered binding energy only in the wild CD6 protein but not in the mutated CD6 at R225W (Table 5). Plastoquinone 8, solamargine, saponin D, and matesaponin 2 decreased energy binding affinity in mutated CD6 proteins at R225W (Table 5). Glycyrrhizin decreased the energy for binding affinity (−11.7 kcal/mol) in wild CD6 proteins at R225W (rs11230563) but not the mutated one (Figure 4A–F).

**Figure 5:**
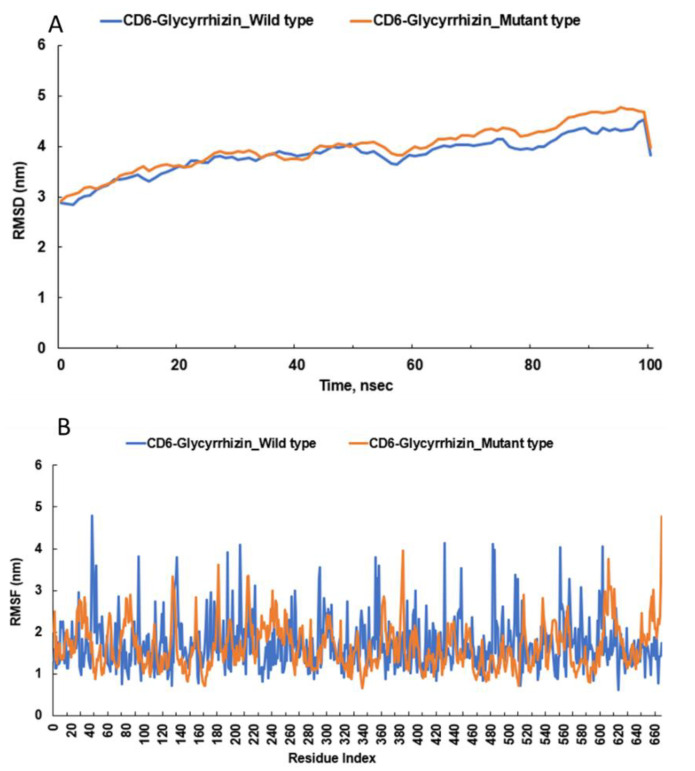
Molecular dynamic simulations between the active site of wild and mutated CD6 protein and glycyrrhizin (**A**) The root mean square deviation (RMSD) of glycyrrhizin binding to the wild and mutated types of CD6. (**B**) The root mean square fluctuations (RMSF) of glycyrrhizin binding to the wild and mutated types of CD6.

RMSD for glycyrrhizin was lower in the wild type of CD6 than in the mutated type (Figure 5A), although it was about 3 nm and within 2.9–3.5 nm over a period of 100 s in both wild and mutated types of CD6. RMSF for glycyrrhizin fluctuated within 0.5–4.3 nm in the wild type and 0.6–3.7 nm in the mutated type (Figure 5B). The results showed that glycyrrhizin had a better binding affinity to the wild type of CD6 than the mutated one. Therefore, people with wild or mutated CD6 proteins should consume appropriate foods to decrease energy-binding affinity. The RMSD and RMSF of azaspiracid 2 in wild type and mutated CD6 rs11230563 (R225W) during molecular dynamic simulation (Figure S1).

### 3.7. Interaction of PRS with Lifestyle Factors Influences DU Risk

**Figure 6:**
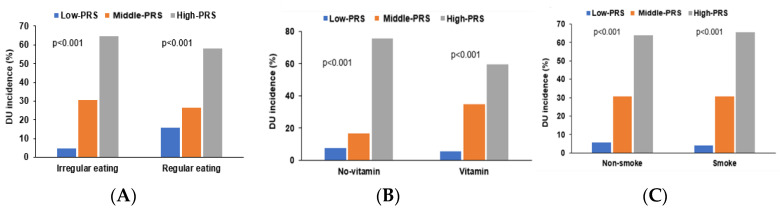
Percentage of the participants with duodenal ulcers according to polygenic risk scores (PRS) in different lifestyles. (**A**) Irregular meal eating. (**B**) Taking multivitamins. (**C**) Smoking status.

DU was associated with lifestyle factors, such as irregular meals, eating raw or burnt meat, WSD, taking multivitamins, and smoking. The interaction between PRS and the lifestyles associated with DU risk was examined. Irregular meals were statistically correlated with DU risk (*p* = 0.0047; Table 6). The DU incidence showed significant differences in participants with irregular eating habits according to the PRS groups. Participants with irregular meal eating habits in the High-PRS exhibited a positive association with DU risk but not those with regular eating habits (Figure 6A). The smoking status also interacted with PRS (*p* = 0.0015; Table 6). DU incidence in the Low-PRS group was lower in current smokers than in non-smokers and former smokers. The PRS showed a positive relation with DU risk in smokers and non-smokers, while the positive association was greater in smokers than in non-smokers (Table 6). In participants taking no multivitamins, the DU incidence was much higher in those with High-PRS than those with Low-PRS and Middle-PRS (Figure 6B). However, the increased DU incidence in the High-PRS group was lower in the participants taking multivitamin (Table 6). WSD (*p* = 0.550), eating raw meat (*p* = 0.623), and eating burnt meat (*p* = 0.780) did not show an interaction with PRS (Figure 6C).

The polygenic risk score (PRS) for the best model was generated by summing the number of the risk alleles in each selected genetic variant with the gene-gene interaction model. The PRS of the model was divided into three categories as Low-PRS (PRS < 4), Middle-PRS (PRS = 4 and 5), and High-PRS (PRS > 5). The adjustment with covariates of age, gender, education, income, residence area, energy intake, alcohol intake, regular exercise, and smoking status.

## 4. Discussion

The stomach secretes acids and enzymes for digestion and to destroy microorganisms. The gastric mucus secreted by the cells in the stomach wall is a barrier to protect the wall from the acid and digestive enzymes within the stomach lumen. A similar mucosal lining protects the duodenal wall [2]. The mucus itself is made up of macromolecules called mucins. There are several well-known causes of DU. The most common causes of DU include inflammation of the duodenum lining due to *H. pylori* infection and prostaglandin inhibition by NSAIDs affecting the regeneration and function of the duodenal mucosal cell layer. Therefore, a decrease in immune function and insufficient mucin are believed to be the key inducers of DU [3]. Genetics and the environment regulate immunity and mucin content in the duodenum. Gastric acid secretion increases with smoking, excessive alcohol consumption, irregular food intake, and mental stress [31]. In the present study, we found that genetic variants associated with DU risk were related to regulating immunity and actin modification [31]. The 5-SNP model included *TTC33*_rs10055925, *FUT2*_rs796980537, *LINC01475*_rs6584283, *ITPR2*_ rs7309887, and *PSCA*_rs2978977. The PRS of the 5-SNP model was positively related to DU risk. Irregular eating, taking multivitamins, and smoking interacted with PRS to influence DU risk. The rs11230563 (R225W) showed a low binding affinity with different food components in the Autodock analysis. Glycyrrhizin, physalin B, janthitrem F, and casuarinin lowered it only in wild CD6 proteins but not in mutated CD6 proteins. Plastoquinone 8, solamargine, saponin D, and matesaponin 2 decreased energy binding affinity in mutated CD6 proteins. These results can be used to devise personalized nutrition for DU prevention.

The genetic polymorphisms involved in DU risk were associated with actin modification genes for the development and differentiation of the intestinal cells and host immunity potentially against *H. pylori* infections [10]. Many studies have implicated *H. pylori* as a significant etiologic factor in DU. Some studies also link *H. pylori* infections with gastric cancer. Thus, in adults, a high host immunity can protect against these disease conditions [32]. In the UK Biobank, *ABO*, *CDX2*, *CCKBR* *MUC1*, *MUC6*, *FUT2*, *PSCA*, and *GAST* genes have been identified and found to be associated with peptic ulcer disease. These genes are believed to be involved in the susceptibility to *H. pylori* infection, protection against infection, gastrointestinal motility, or gastric acid secretion [10]. Genetic polymorphisms, including *FUT2* and *PSCA*, are also associated with DU, consistent with the present study. The genetic predisposition of disintegrin and metalloproteases (*ADAMs*) and Th17-related cytokines, such as *IL-17A*, *IL-17F*, *IL-33*, *IL-23*, and *IL-23R*, was associated with DU risk [10,33]. Similar to earlier studies, the genetic variants of *IL1RN, IL23R*, and *IL1R2* were found in the present study, but their statistical significance did not reach *p* < 5 × 10^−8^. Therefore, poor immunity, potentially to *H. pylori*, is linked to DU risk.

DU is associated with damage to the duodenal wall due to vigorous immune responses or severe cellular inflammatory responses. The genetic polymorphisms of proinflammatory cytokines, such as *TNF-α* [34], *IL-1β*, *IL-4*, and *IL-8*, and related to *H. pylori* infection, have been seen in different ethnicities [35,36]. The present study selected a genetic variant of lipopolysaccharide-induced TNF factor (*LITAF*) for DU risk among the genetic variants related to proinflammatory cytokines. While some genetic polymorphisms of IL were associated with DU risk, they did not meet the statistical significance of *p* < 5 × 10^−6^. A few studies have shown the association of IL genetic polymorphisms with DU risk in Koreans [37], which are reported to be associated with gastric cancer in Asian subjects [38,39]. *LITAF* acts as a mediator of local and systemic inflammatory responses to modulate the increment of TNF-α secretion in inflammatory diseases, including irritable bowel disease [40,41]. Lipopolysaccharides (LPS) are the major outer surface membrane components of *H. pylori* and act as potent stimulators of the host immune response [41]. An *H. pylori* infection increases *TNF-α* expression with *LITAF.* Therefore, *H. pylori* infection is associated with DU risk by modulating immunity and LPS-related inflammation.

Prostate stem cell antigen (*PSCA*), a glycosylphosphatidylinositol-anchored cell membrane glycoprotein, is involved in not only prostate cancer but also gastric cancer and DU in different ethnicities [2,11]. *PSCA* s2978977 is not linked to GU in Japanese, consistent with the present study [11]. However, the T allele of *PSCA* rs2978977 has been shown to be associated with gastric cancer (OR = 1.109, *p* = 8.11 × 10^−10^) and an inverse association with DU (OR = 0.648, *p* = 6.86 × 10^−19^) in the present study, consistent with previous studies [42,43]. The Cox regression analysis has revealed the T allele of rs2294008 as a prognosis factor in patients with diffuse-type gastric cancer (hazard ratio: 1.85; 95% CI: 1.12–3.06). The fragile histidine triad (*FHIT*) is also linked to protecting against developing *H. pylori*-related gastric cancer. However, the association between *FHIT* expression and DU risk may occur through the prevention or eradication of *H. pylori* infections [44]. *ITPR2* also acts as a second messenger to modulate intracellular calcium levels by releasing it from the endoplasmic reticulum [45]. Intracellular calcium plays a critical role in gastric and duodenal wound repair, and its polymorphism influences DU risk [46]. Therefore, *ITPR2* polymorphism can alter DU risk, and the A allele of rs7309887 might suppress duodenal cell repair in DU.

Although many studies have studied the relationship between genetic variants and diseases, their direct impacts are small and difficult to observe in small epidemiological studies. Genetic polymorphisms alter gene expressions, protein production, and binding affinity to molecular components such as medications and food. Many genetic polymorphisms are in the intron and promoter regions and alter gene expression. However, the mechanisms by which genetic variants in the intron affect the corresponding gene expression have yet to be revealed in the present study. Missense genetic variants can influence gene function by changing binding affinity to components, including food and drugs [47]. The ligand binding site of rs11230563_*CD6* (R225W) missense was selected to find the food components to decrease their binding affinity, which may act as the regulators of the CD6 activity. CD6 is a well-known target protein for regulating immune responses [48]. CD6 negatively and positively influences the initiation and maintenance of T cell function, respectively [48]. Therefore, the mutation in the *CD6* can affect binding affinity to food components by changing free energy. The present study showed that the rs11230563 nucleotide site changed binding affinity to a particular molecular site of the *CD6* gene. These results indicated that rs11230563 affects the domains 1 and 3 areas to alter binding affinity to food components. People with the C allele of rs11230563 *CD6* had a higher chance of inducing DU risk, and the food components to lower the binding affinity of the C allele in rs11230563 in *CD6* might improve CD6 activity. Therefore, glycyrrhizin, physalin B, janthitrem F, and casuarinin might improve CD6 activity due to the lower binding energy in the *CD6* with the C allele in rs11230563. However, the relationship between binding affinity and CD6 function needs to be validated.

The primary risk factor for DU is an *H. pylori* infection, and additional ones are being male, family history, blood type, skipping breakfast or more than one meal, coffee intake, consumption of NSAIDs, aspirin, alcohol, coffee, and cigarette smoking [49]. The present study showed consistent results for all of the above except for coffee intake. Among dietary patterns, DU was negatively associated with WSD but not KBD, PBD, and RMD. Irregular meal intake and eating uncooked and burnt meats were positively linked to DU risk. Exercise, alcohol intake, and mental stress have been reported to be related to DU risk [31], but the association was not seen in the present study. Coffee drinking, taking multivitamin, and smoking status were inversely associated with DU risk in the present study. No study to date has reported that lifestyle modifications interact with PRS. In the present study, the PRS associated with DU risk interacted with irregular meal intake, smoking, and multivitamin intake. The results suggested that people with high-PRS better to consume regular meals, take multivitamins, and smoke less to reduce DU risk.

A few studies have studied the genetic polymorphism of DU risk, although most studies have been conducted on peptic ulcers, including both gastric and duodenal ulcers. To the best of our knowledge, it is novel to determine the interaction between duodenal ulcers and lifestyles in a large hospital-based cohort (n = 58,701). The limitations of the study were (1) the cause-and-effect relationship could not be explained since it was a case–control study. (2) The patient history of a physician-diagnosed duodenal ulcer was brought up by recollection from the memory of the participant, not by the medical record. (3) The usual food intake for the previous six months was assessed using an SQFFQ based on memory. However, the SQFFQ contained 106 food items related to Korean diets and was validated by 3-day food records for four seasons [19].

In conclusion, the PRS of the selected genetic variants for DU risk was mainly associated with actin modification, LRR domain binding, Shaffer IRF4 targets in myeloma vs. mature B lymphocytes, and Reactome runx3 regulated immune response and cell migration. The results suggest that the DU risk was related to modulating immunity, inflammation, and intestinal cell repair. Among the selected genetic variants, rs11230563_*CD6* (R225W), missense mutation, was shown to reduce binding affinity with glycyrrhizin, physalin B, janthitrem F, and casuarinin in the T allele, and with plastoquinone 8, solamargine, saponin D, and matesaponin 2 in the C allele of rs11230563 *CD6* in the Autodock analysis. The High-PRS of the 5–SNP and 6–SNP models showed a positive association with DU risk by about three times. In participants with irregular eating and rare meat-eating habits, DU incidence was much higher in the participants with High-PRS than those with Low-PRS, as with smokers. These results suggest that the genetic impact of DU risk was primarily involved with mediating immunity, inflammation, and actin modification. DU risk was linked to rare meat, irregular eating habits, and smoking status. The interaction of eating habits with PRS for DU risk and the missense mutations of genetic variants can be applied to devise personalized nutrition.

## Figures and Tables

**Table 1:** General characteristics of the participants according to gender and duodenal ulcer.

	Men		Women		Adjusted ORs (95% CI)
	Non-Ulcer (n = 19,725)	DU (n = 540)	Non-Ulcer (n = 37,810)	DU (n = 548)	
Age (years) ^3^	57.1 ± 0.09 ^b 1^	58.3 ± 0.42 ^a^	52.3 ± 0.06 ^c^	53.4 ± 0.45 ^c *** +++^	1.40 (1.22–1.61) ^2^
Gender (%)	36.1	49.1 ^⁑⁑⁑^	63.9	50.9 ^⁑⁑⁑^	0.686 (0.570–0.827)
BMI (kg/m^2^) ^4^	24.5 ± 0.03 ^a^	23.8 ± 0.16 ^b^	23.5 ± 0.02 ^bc^	23.4 ± 0.17 ^c *** +++^	0.777 (0.670–0.900)
Waist circumferences (cm) ^5^	85.3 ± 0.07 ^a^	84.1 ± 0.35 ^b^	78.3 ± 0.05 ^c^	77.8 ± 0.35 ^c *** ++^	0.781 (0.657–0.928)
Serum glucose (mg/dL) ^6^	97.7 ± 0.19 ^a^	94.9 ± 0.91 ^b^	93.8 ± 0.12 ^b^	92.9 ± 0.90 ^b *** ++^	0.796 (0.628–1.009)
Blood HbA1c (%) ^7^	5.68 ± 0.01 ^b^	5.56 ± 0.05 ^b^	5.72 ± 0.01 ^a^	5.78 ± 0.05 ^a *** #^	0.720 (0.506–1.025)
WBC (10^9^/L) ^8^	5.76 ± 0.02 ^b^	5.84 ± 0.09 ^a^	5.67 ± 0.01 ^c^	5.47 ± 0.09 ^d ***^	0.753 (0.657–0.863)
Serum hs-CRP (mg/dL) ^9^	0.14 ± 0.004	0.13 ± 0.02	0.14 ± 0.002	0.13 ± 0.02	0.794 (0.436–1.448)
MetS (*n*, Yes%)	3503 (17.8)	88 (16.3)	4625 (12.2)	71 (13.0)	0.900 (0.748–1.083)
Bronchitis (*n*, Yes%)	135 (0.61)	23 (1.85) ^⁑⁑^	257 (0.62)	15 (1.83) ^⁑⁑^	2.42 (1.38–4.25)
Asthma (*n*, Yes%)	261 (1.38)	16 (3.14) ^⁑⁑^	630 (1.77)	18 (3.53) ^⁑⁑^	2.17 (1.50–3.13)
Arthritis (*n*, %)	727 (3.85)	39 (7.65) ^⁑⁑⁑^	3886 (10.9)	110 (21.5) ^⁑⁑⁑^	2.19 (1.80–2.67)
Allergy (*n*, %)	1011 (5.53)	53 (10.4) ^⁑⁑⁑^	2716 (7.64)	73 (14.3) ^⁑⁑⁑^	2.06 (1.68–2.53)
Gastritis (*n*, %)	1438 (7.61)	102 (20.0) ^⁑⁑⁑^	3720 (10.5)	152 (29.8) ^⁑⁑⁑^	3.34 (2.86–3.91)
Periodontitis (*n*, Yes%)	1394 (7.38)	74 (14.5) ^⁑⁑⁑^	2221 (6.25)	68 (13.3) ^⁑⁑⁑^	2.18 (1.79–2.67)
Osteoporosis (*n*, Yes%)	123 (0.62)	9 (1.67) ^⁑⁑^	2613 (7.35)	70 (13.7) ^⁑⁑⁑^	1.93 (1.49–2.50)

^1^ Values represented adjusted means ± standard error for continuous variables and the number and percentages of participants for categorical variables. ^2^ Adjusted odds ratio (OR) and 95% confidence intervals (CI). Covariates included age, sex, education, income, energy intake (percentage of estimated energy requirement), residence areas, daily activity, alcohol intake, and smoking status. The cutoff points of the reference for logistic regression are as follows: ^3^ <55 years; ^4^ <25 kg/m^2^ for body mass index (BMI); ^5^ <90 cm for men and 85 cm for women waist circumferences; ^6^ <126 mL/dL fasting serum glucose plus diabetic drug intake; ^7^ <6.5% hbA1c plus diabetic drug intake; ^8^ <4.0 × 10^9^/L white blood cells (WBC); ^9^ <1.0 mg/dL high-sensitive C-reactive protein (hs-CRP). ^***^ Significant differences by genders at *p* < 0.001. ^++^ Significant differences by DU at *p* < 0.01, ^+++^ *p* < 0.001. ^#^ Significant interaction between genders and DU at *p* < 0.05. ^⁑⁑^ Significantly different from the control group in χ^2^ test in each gender at *p* < 0.01, ^⁑⁑⁑^ at *p* < 0.001. a, b, c, d Different superscripts on bars indicate significant differences among the groups at *p* < 0.05.

**Table 2:** Dietary intake and lifestyles according to gender and duodenal ulcer.

	Men		Women		Adjusted ORs (95% CI)
	No-Ulcer (n = 19,725)	Ulcer (n = 540)	No-Ulcer (n = 37,810)	Ulcer (n = 548)	
Energy intake (kcal/day) ^3^	90.0 ± 0.38 ^b 1^	90.1 ± 1.82 ^b^	101 ± 0.27 ^a^	102 ± 1.93 ^a ***^	1.011 (0.824–1.239) ^2^
KBD (N, Yes%) ^4^	7852 (40.1)	209 (41.0)	10,686 (30.1)	143 (27.9)	0.989 (0.850–1.150)
PBD (N, Yes%) ^4^	3833 (20.3)	103 (20.2)	14,151 (39.8)	206 (40.2)	1.022 (0.874–1.196)
WSD (N, Yes%) ^4^	9968 (52.7)	230 (45.1) ^⁑⁑^	12,619 (35.5)	155 (30.3) ^⁑^	0.809 (0.695–0.941)
RMD (N, Yes%) ^4^	6019 (31.8)	162 (31.8)	12,131 (34.1)	159 (31.1)	0.918 (0.791–1.064)
Irregular meals	67 (1.13)	3 (1.58)	232 (2.22)	9 (4.57) ^⁑^	1.965 (1.029–3.751)
Less cooked meats (N, Yes%)	11,792 (62.5)	336 (66.1) ^⁑^	18,788 (53.0)	302 (59.1) ^⁑⁑^	1.243 (1.082–1.428)
Burnt meats (N, Yes%)	3493 (19.3)	101 (20.7)	4371 (12.6)	72 (14.4)	1.197 (1.001–1.431)
Fried foods (N, Yes%) ^5^	11,452 (60.6)	294 (57.7)	23,312 (65.6)	345 (67.4)	0.940 (0.817–1.081)
Coffee (g/day) ^6^	3.65 ± 0.03 ^a^	3.28 ± 0.14 ^b^	3.69 ± 0.02 ^a^	3.03 ± 0.13 ^b +++^	0.648 (0.567–0.740)
Tea (g/day) ^7^ 45	43.9 ± 0.81	40.5 ± 3.89	42.9 ± 0.53	45.9 ± 3.84	1.115 (0.959–1.297)
Alcohol (g/day) ^8^	30.6 ± 0.64 ^a^	30.2 ± 3.05 ^a^	9.13 ± 0.45 ^b^	9.09 ± 3.23 ^b ***^	1.019 (0.880–1.179)
Multivitamin (N, Yes%)	14,647 (74.3)	378 (70.0) ^⁑⁑^	29,158 (77.1)	396 (72.3) ^⁑⁑^	0.779 (0.673–0.903)
Physical activity (N, Yes%)	11,611 (59.0)	330 (61.5)	19,725 (52.3)	287 (52.7)	1.451 (0.201–10.46)
Former smoker (N, %)	8515 (43.3)	272 (50.5) ^⁑⁑⁑^	449 (1.19)	11 (2.01)	1.516 (1.220–1.883)
Current smoker (N, %)	5501 (28.0)	157 (29.1)	737 (1.96)	12 (2.19)	1.475 (1.162–1.872)

^1^ Values represented adjusted means ± standard error for continuous variables and the number and percentages of participants for categorical variables. ^2^ Adjusted odds ratio (OR) and 95% confidence intervals. Covariates included age, sex, education, income, energy intake (percentage of estimated energy requirement), residence areas, daily activity, alcohol intake, and smoking status. The cutoff points of the reference for logistic regression are as follows: ^3^ <Estimated energy requirement (EER); ^4^ <25th percentiles of each dietary pattern; ^5^ <twice/week; ^6^ <3 g/day; ^7^ <45 g/day; ^8^ <20 g/day. ^***^ Significant differences by genders at *p* < 0.001. ^+++^ Significant differences by DU at *p* < 0.001. ^⁑^ Significantly different from the control group in χ^2^ test in each gender at *p* < 0.05, ^⁑⁑^ at *p* < 0.01, and ^⁑⁑⁑^ at *p* < 0.001. a, b Different superscripts on bars indicate significant differences among the groups at *p* < 0.05.

**Table 3:** Characteristics of ten genetic variants selected for their interactions by GMDR.

CHR	SNP	BP	A1	A2	OR	SE	*p*	MAF	HWE_P	Gene Names	Functional SEQUENCES
2	rs576376935	218,990,311	G	T	1.945	0.159	2.88 × 10^−6^	0.0113	0.3496	*CXCR2*	Intron
3	rs77063016	60,431,863	C	G	1.314	0.06484	2.52 × 10^−6^	0.1183	0.8432	*FHIT*	Intron
5	rs10055925	40,688,059	A	G	0.7662	0.04673	1.21 × 10^−8^	0.4778	0.0509	*TTC33*	Intron
8	rs2978977	143,755,720	A	C	0.6365	0.0509	6.86 × 10^−19^	0.3857	0.5421	*PSCA*	Intron
10	rs6584283	101,290,301	T	C	1.204	0.04625	4.13 × 10^−6^	0.4633	0.7842	*LINC01475*	Intron
11	rs11230563 (R225W)	60,776,209	T	C	0.808	0.06226	4.18 × 10^−6^	0.1932	0.7708	*CD6*	Missense
12	rs7309887	26,583,100	C	A	0.8127	0.04997	3.32 × 10^−6^	0.3536	0.7112	*ITPR2*	NMD transcript
13	rs78141015	43,664,299	T	C	1.702	0.1124	2.21 × 10^−6^	0.0276	0.7582	*DNAJC15*	Intron
16	rs111690253	11,688,746	T	A	1.914	0.1251	2.12 × 10^−7^	0.0192	0.1524	*LITAF*	Intron
19	rs796980537	49,203,590	T	A	0.7424	0.0753	6.03 × 10^−6^	0.1359	0.1003	*FUT2*	Intron

*PSCA*, prostate stem cell antigen; *FHIT*, fragile histidine triad diadenosine triphosphatase; *CD6*, cluster of differentiation 6; *ITPR2*, inositol 1,4,5-trisphosphate receptor type 2; *TTC33,* tetratricopeptide repeat domain 33; *FUT2*, fucosyltransferase 2; *DNAJC15*, DnaJ heat shock protein family; *LITAF*, lipopolysaccharide-induced TNF factor; *CXCR2*, C-X-C Motif Chemokine Receptor 2.

**Table 4:** Pathways related to genetic variants for duodenal ulcer risk.

Pathways	No. of Genes	Beta	SD	*p* Value Bonferroni	Participating Genes
GO BP: GO Actin modification	5	1.6621	0.027085	6.6387 × 10^−5^	*CXCR2, FHIT, CD6, ITPR2, DNAJC15*
GO MF: GO LRR domain binding	17	0.72595	0.021805	9.8491 × 10^−5^	*CD6, FUT2*, *FHIT*
Curated gene sets: Shaffer IRF4 targets in myeloma vs. mature B lymphocyte	96	0.30049	0.021404	0.00014762	*CXCR2, CD6, ITPR2, FUT2*
Curated gene sets: Reactome runx3 regulates immune response and cell migration	6	1.2508	0.022326	0.00019992	*CXCR2, FHIT, CD6, ITPR2, DNAJC15*

SD, standard deviation; GO, gene ontology; BP, biological process; MF, molecular function; IRF4, Interferon Regulatory Factor 4; runx3, runt-related transcription factor 3.

**Table 5:** The binding affinity of food compounds with wild and mutated *CD6* proteins measured by free energy changes (ΔG; kcal/mol).

Active Ingredients	Effective Food	ΔG of wild CD6	ΔG of Mutant CD6
Azaspiracid 2	Blue mussel	−13.2	−13.2
Glycyrrhizin	Liquorice	−11.7	-
Physalin B	Winter cherry	−12.4	-
Janthitrem F	Penicillium Janthinellum	−12	-
Casuarinin	Siberian filbert	−11.5	-
Plastoquinone 8	Sweet corn	-	−12.3
Solamargine	Solanaceae family	-	−12.2
Saponin D	Hovenia dulcis	-	−11.2
Matesaponin 2	Ilex paraguariensis	-	−11.9

−, less than −10 of ΔG.

**Table 6:** Adjusted odds ratios for the duodenal ulcer risk by polygenetic risk scores of the best model (PRS) after covariate adjustments according to the patterns of lifestyles.

	Low-PRS (n = 5912)	Middle-PRS (n = 23,471)	High-PRS (n = 29,240)	Interaction
Irregular meal	1	2.237 (1.196–4.185)	3.674 (1.996–6.762)	0.0047
Regular meal	1	0.062 (0.003–1.012)	0.742 (0.115–4.773)	
Non-smoking + former	1	1.718 (0.934–3.161)	3.041 (1.689–5.475)	0.0015
Smoking	1	4.285 (0.566–32.43)	5.301 (0.713–39.38)	
No multivitamin	1	2.215 (1.146–4.278)	3.057 (1.606–5.821)	0.0055
Multivitamin	1	1.016 (0.287–3.600)	3.789 (1.174–12.23)	

## Data Availability

The raw data involved in this study will be available by the corresponding author to any qualified researcher.

## Supplementary Materials

The following supporting information can be downloaded at: https://www.mdpi.com/article/10.3390/nu15020296/s1, Table S1. Generalized multifactor dimensionality reduction (GMDR) results of multi-locus interaction with genes related to duodenal ulcer risk. Table S2. The adjusted odds ratio of polygenic risk scores with duodenal ulcer risk. Figure S1. The root mean square deviation (RMSD) and root mean square fluctuations (RMSF) of azaspiracid 2 (A and B) in wild-type and RMSD and RMSF of azaspiracid 2 (C and D) in mutated CD6 rs11230563 (R225W) during molecular dynamic simulation.
