# Supplementary material for: Association of Polygenic Variants Involved in Immunity and Inflammation with Duodenal Ulcer Risk and Their Interaction with Irregular Eating Habits
Source: Nutrients. 2023 Jan 6;15(2):296. doi: 10.3390/nu15020296 (PMC9863374; doi:10.3390/nu15020296)
Supplement: Supplementary file 1 [file nutrients-15-00296-s001.zip › nutrients-2122876-supplementary.pdf]

Supplementary Table S1. Generalized multifactor dimensionality reduction (GMDR) results of multi-locus interaction with genes related to duodenal ulcer risk.

	Model 1				Model 2			
	TRBA	TEBA	P value	CVC	TRBA	TEBA	P value	CVC
*PSCA*_rs2978977	0.5692	0.5701	0.001	10	0.5692	0.5701	0.001	10
*FHIT*_rs77063016 plus model 1	0.5806	0.5803	0.001	10	0.5806	0.5803	0.001	10
*CD6*_rs11230563 plus model 2	0.5873	0.5781	0.001	8	0.5873	0.5781	0.001	8
*ITPR2*_rs7309887 plus model 3	0.5972	0.5596	0.001	3	0.5972	0.5596	0.001	3
*TTC33*_rs10055925 *FUT2*_rs1047781 *LINC01475*_rs6584283 *ITPR2*_rs7309887 *PSCA*_rs2978977	0.6214	0.5617	0.001	10	0.6214	0.5617	0.001	10
*CD6*_rs11230563 plus model 5	0.6602	0.5566	0.001	10	0.6602	0.5566	0.001	10
*FHIT*_rs77063016 plus model 6	0.7069	0.5402	0.001	10	0.7069	0.5402	0.001	10
*DNAJC15*_rs78141015 plus model 7	0.7259	0.5352	0.001	10	0.7259	0.5352	0.001	10
*LITAF*_rs111690253 plus model 8	0.7404	0.545	0.001	10	0.7404	0.545	0.001	10
*CXCR2*_rs576376935 plus model 9	0.7496	0.5425	0.001	10	0.7496	0.5425	0.001	10

TRBA, training balanced accuracy; TEBA, testing balanced accuracy; CVC, cross-validated consistency; P-value for assessing the best model. *PSCA*, prostate stem cell antigen; *FHIT*, fragile histidine triad diadenosine triphosphatase; *CD6*, cluster of differentiation 6; *ITPR2*, inositol 1,4,5-trisphosphate receptor type 2; *TTC33,* tetratricopeptide repeat domain 33; *FUT2*, fucosyltransferase 2; *DNAJC15*, DnaJ heat shock protein family; *LITAF*, lipopolysaccharide induced TNF factor; *CXCR2*, C-X-C Motif Chemokine Receptor 2. Covariates for model 1: sex, residence area, age, weight, education, and income. Covariates for model 2: covariates for model 1 plus smoking, energy intake, alcohol intake and physical exercise.

Supplementary Table S2. Adjusted odds ratio of polygenic risk scores with duodenal ulcer risk

	Low-PRS (n=5912)	Medium-PRS (n=23,471)	High-PRS (n=29,240)	Adjusted OR
WBC (10^9/L)	5.74±0.02	5.69±0.01	5.71±0.01	0.996 (0.958-1.036)
CRP (mg/dL)	0.13±0.006	0.14±0.003	0.14±0.003	0.963 (0.822-1.129)
BMI (kg/m^2)	23.9±0.04	23.9±0.02	23.9±0.02	1.05 (1 1.009-1.094)
Waist (cm)	808±0.11	80.6±0.05	80.7±0.05	1.009 (0.963- 1.058)
Metabolic syndrome (N, Yes %)	802 (14.2)	3051 (13.7)	3901 (14.1)	1.047 (0.991- 1.107)
Bronchitis (N, Yes %)	43 (7.6)	173 (7.8)	212 (7.7)	1.107 (0.871- 1.408)
Asthma (N, Yes %)	84 (1.49)	373 (1.68)	468 (1.70)	0.992 (0.859- 1.145)
Arthritis (N, %)	476 (8.45)	1893 (8.52)	2393 (8.67)	1.007 (0.939- 1.080)
Allergy (N, %)	375 (6.64)	1577 (7.10)	1902 (6.89)	0.970 (0.901- 1.044)
Osteoporosis (N, Yes%)	269 (4.78)	1135 (5.11)	1404 (5.09)	0.984 (0.899- 1.076)
Gastritis (N, %)	540 (9.59)	2128 (9.58)	2744 (9.94)	1.041 (0.977- 1.109)
Periodontitis (N, Yes%)	365 (6.48)	1414 (6.37)	1752 (6.35)	1.009 (0.934- 1.089)

The polygenic risk score (PRS) for the best model was generated by summing the number of the risk alleles in each selected genetic variant with the gene-gene interaction model. The PRS of the model was divided into three categories as Low-PRS (PRS<3), Middle-PRS (PRS=3 and 4), and High-PRS (PRS>4). The adjustment with covariates of age, gender, weight at 18 years old, education, income, and residence area, energy intake, alcohol intake, regular exercise, and smoking status.

Supplementary Figure

Figure S1. The root mean square deviation (RMSD) and root mean square fluctuations (RMSF) of azaspiracid 2 in wild type and mutated CD6 rs11230563 (R225W) during molecular dynami[illegible] simulation.

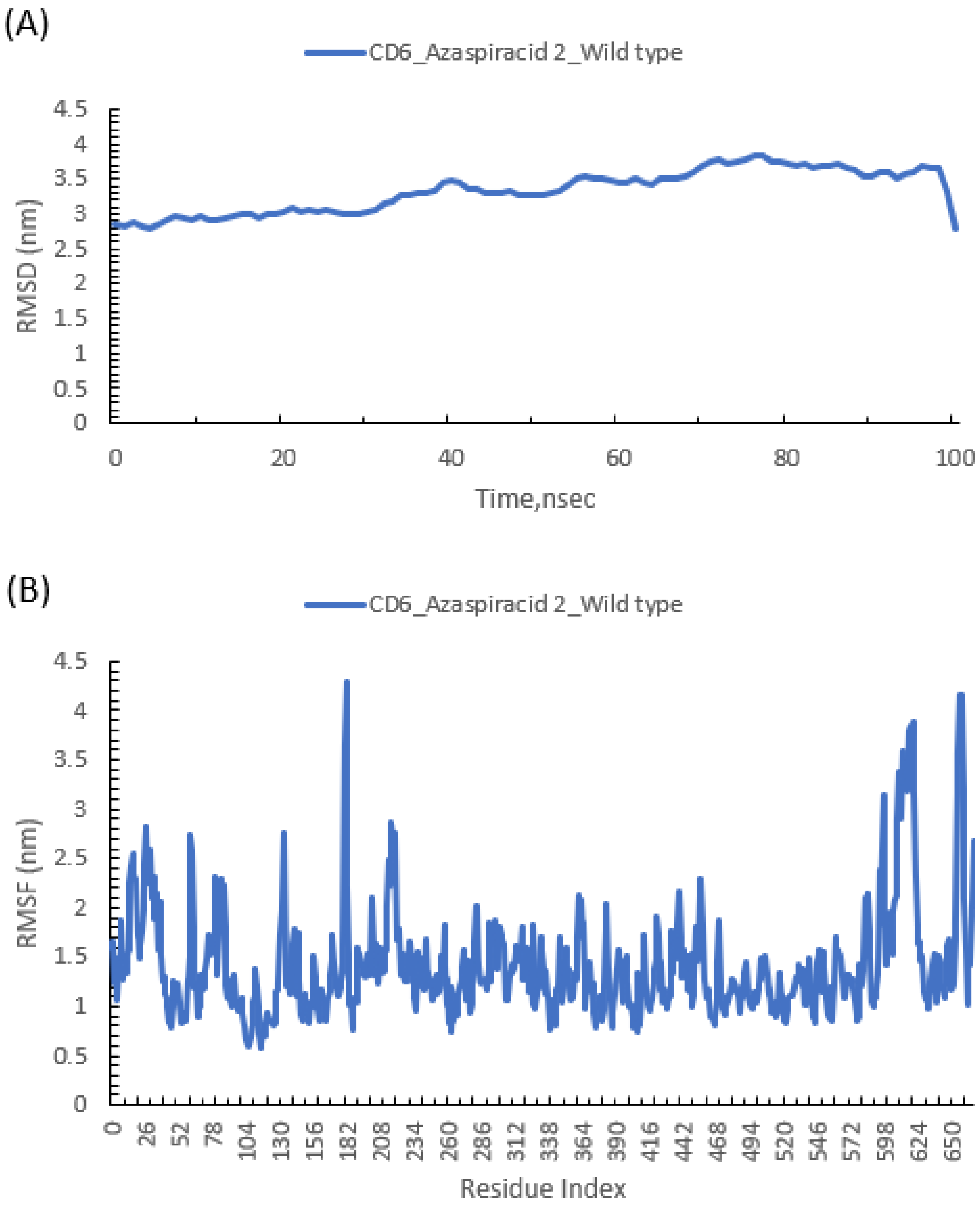

(C)

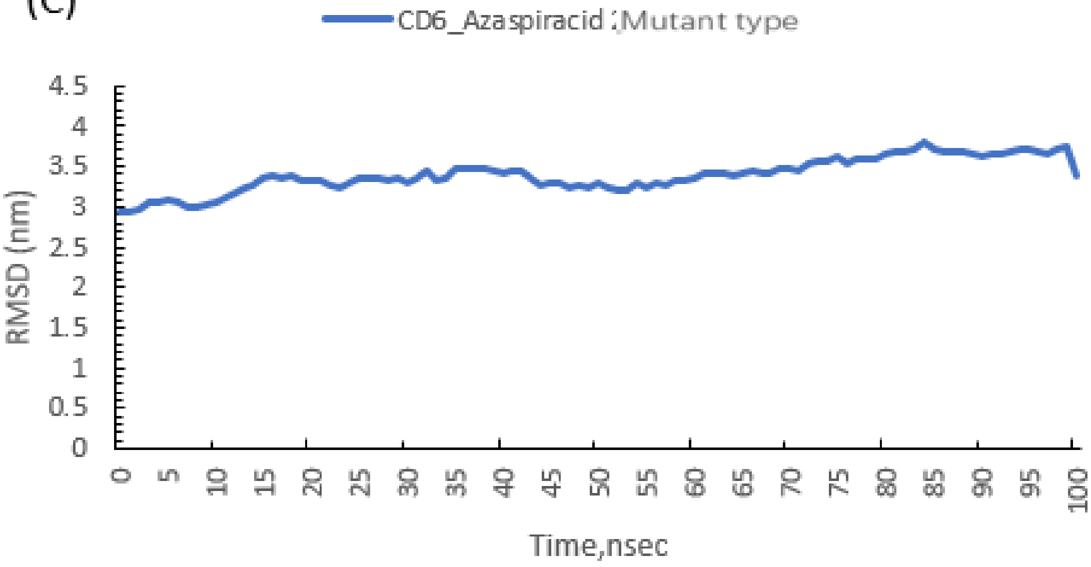

CD6_Azaspiracid 2_Mutant type
RMSD (nm)
Time,nsec

(D)

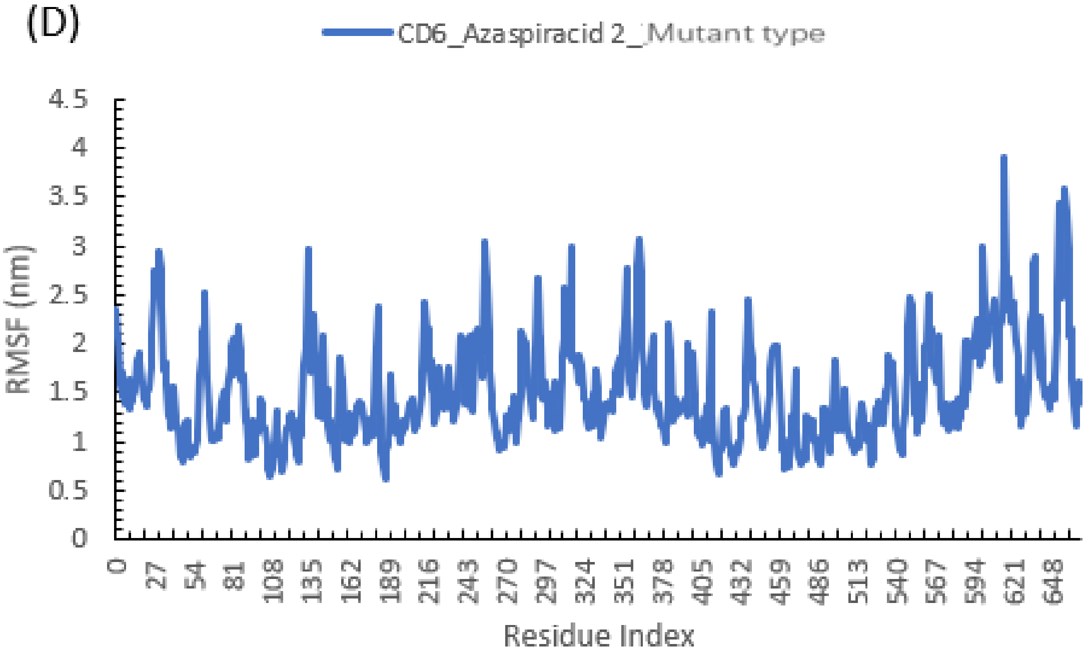

CD6_Azaspiracid 2_Mutant type
RMSF (nm)
Residue Index
